# Copper Levels in Patients with Unexplained Dysplastic Cytopenia

**DOI:** 10.1007/s12011-020-02203-x

**Published:** 2020-06-02

**Authors:** Dominic Butcher, Simona Deplano, Thomas Lofaro

**Affiliations:** 1 Department of Medicine, Imperial College London, South Kensington, London, UK; 2 Imperial College NHS Healthcare Trust, Department of Haematology, Hammersmith Hospital, Du Cane Road, London, UK

**Keywords:** Copper, Myelodysplasia, MDS, Cytopenia, Dysplasia, Hypocupremia

## Abstract

Copper deficiency can produce changes mimicking myelodysplasia, but its prevalence among haematology patients is unknown. This study evaluates the prevalence of deficiency in a population of patients with unexplained cytopenia(s) and dysplasia. Of 17 patients recruited, only one was found to have a low copper level. This is the first study evaluating hypocupremia in patients with isolated haematological manifestations. Our results suggest that copper deficiency is uncommon in this population, and the routine measurement of copper seems unlikely to be cost-effective. We recommend a risk-assessed approach to testing based on the identification of risk factors and of signs suggestive of deficiency.

**Electronic supplementary material:**

The online version of this article (10.1007/s12011-020-02203-x) contains supplementary material, which is available to authorized users.

## Introduction

**Fig. 1:**
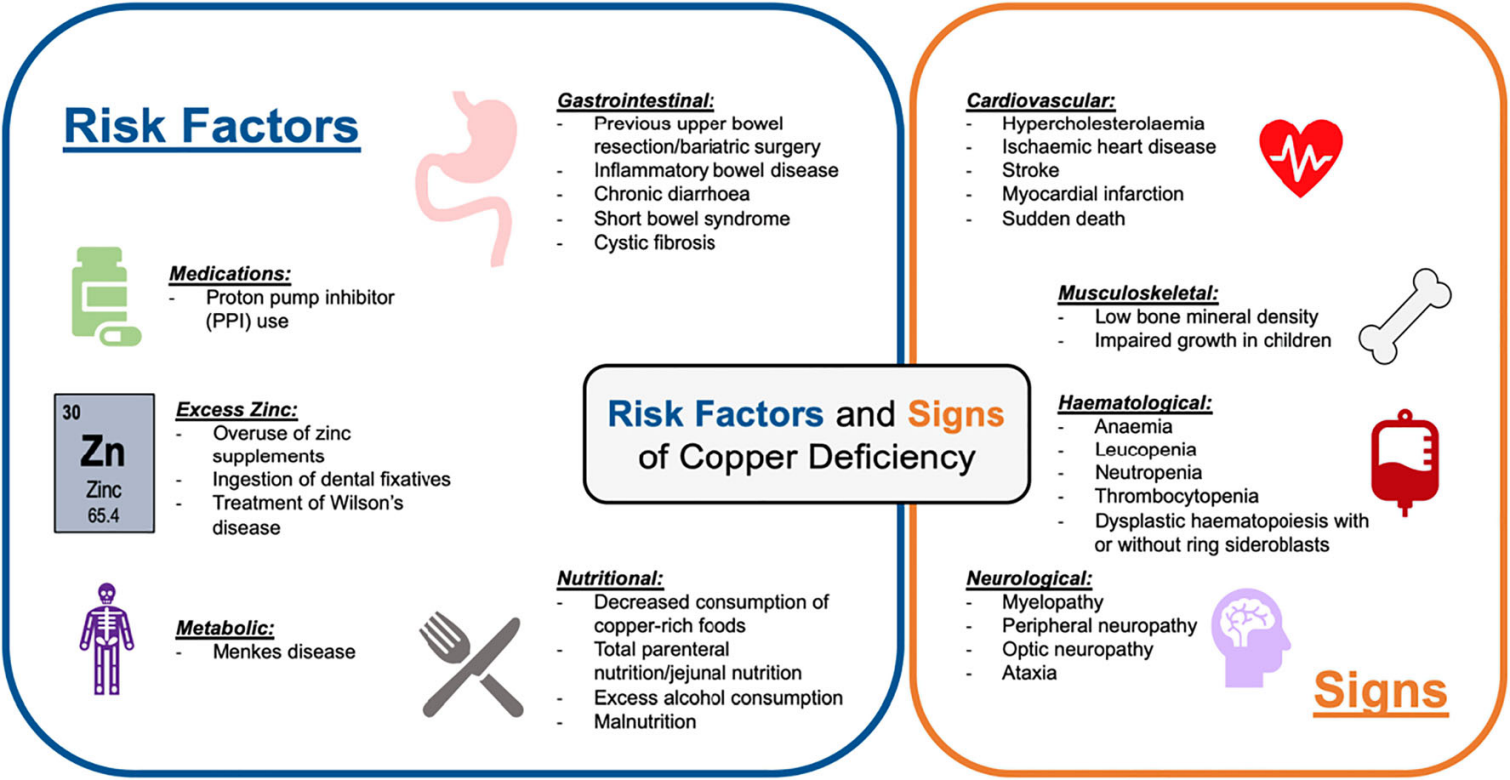
The risk factors for and signs of copper deficiency. Please see references: risk factors [1, 2, 4–11] and signs [1, 2, 9, 12, 13]

Copper is an essential micronutrient that is incorporated into copper-dependent enzymes and plays an integral role in oxidation-reduction reactions. Several risk factors for copper deficiency are reported in the literature, and hypocupremia can present with a variety of metabolic, haematological and neurological manifestations (Fig. 1). To the haematologist, copper deficiency can present with signs of bone marrow failure and dysplastic haematopoiesis [1, 2], and can be misdiagnosed as myelodysplastic syndrome (MDS) [3].

There is uncertainty regarding the role of copper measurement in patients with unexplained cytopenia. The prevalence of copper deficiency among unselected patients is unknown [6], and the only data available is in select groups of patients [6, 14]. Myelodysplasia (MDS) is an important differential of copper deficiency, and is characterised by cytopenia(s), dysplastic haematopoiesis and an increased risk of transformation to acute leukaemia. Under the microscope, copper deficiency may uniquely produce vacuolation in the myeloid precursors, and this finding should prompt consideration for this differential. Pelger-Huet neutrophils, dysmegakaryopoiesis and an excess of blasts, on the other hand, favour a diagnosis of MDS [15].

Cytogenetic and molecular analysis are able to demonstrate evidence of clonal haematopoiesis in 75% of patients with MDS [16], and this raises the question of whether other pathology may be present in those patients in whom no clonal marker is found. Possible precursor conditions for MDS also need to be considered in the differential diagnosis, including idiopathic cytopenia of uncertain significance (ICUS) and idiopathic dysplasia of uncertain significance (IDUS). ICUS is characterised by persistent unexplained cytopenia of more than 6 months’ duration, with dysplastic features absent or only visible in less than 10% of cells in each cell line. IDUS is a condition with dysplastic features in excess of 10% in one or more cell lines but without significant cytopenia, and with or without genetic or molecular markers of clonality.

MDS should only be diagnosed when other causes of the haematological features have been excluded. A diagnosis of MDS has significant implications for patients, not least because of the known risk of progression to leukaemia. If an alternative cause is missed, patients will be subjected to needless investigations and hospital appointments, and will also be at risk of developing complications of their condition that could have been prevented with appropriate treatment.

We designed a study to evaluate the prevalence of copper deficiency in patients with cytopenias and dysplasia who also lack a cytogenetic or molecular marker of MDS. The participants were all patients who were being monitored in our haematology clinic. The majority had bone marrow dysplasia reported in excess of 10%, and therefore fulfilled WHO criteria for a diagnosis of myelodysplasia.

## Methods

We evaluated our clinic and bone marrow records to identify potential participants. We wanted to exclude patients in whom a true diagnosis of MDS was highly likely. To achieve this, we excluded patients with a confirmed cytogenetic or molecular abnormality (with the exception of deletion of chromosome Y), those with a marrow blast percentage in excess of 5% on cytological examination or immunophenotyping, and patients who were receiving (or had previously received) cytotoxic treatment for MDS.

We identified 17 potential patients who met our inclusion criteria (ESM 1). Eligible patients were offered information about the study and invited to participate. All 17 patients gave consent. As part of the study, patients were asked to complete a health questionnaire (ESM 6) and to submit a blood test for measurement of their serum copper level. The questionnaire was designed to identify risk factors for the development of copper deficiency and included questions about diet, drug history, the use of mineral supplements, gastrointestinal pathology, bowel surgery, dialysis and alcohol intake. Additional questions were included to identify unexpected symptoms of the condition.

Mutation screening in our centre is performed by targeted next generation sequencing using TruSight Myeloid panel (Illumina) using the Qiagen QIAsymphony DSP DNA Mini Kit. This assay comprises of 568 amplicons in 54 genes frequently mutated in myeloid malignancies, including mutations in *ABL1*, *ASXL1*, *ATRX*, *BCOR*, *BCORL1*, *BRAF*, *CALR*, *CBL*, *CBLB*, *CBLC*, *CDKN2A*, *CSF3R*, *CEBPA*, *CUX1*, *DNMT3A*, *ETV6*, *EZH2*, *FBXW7*, *FLT3*, *GATA1*, *GATA2*, *GNAS*, *HRAS*, *IDH1*, *IDH2*, *IKZF1*, *JAK2*, *JAK3*, *KDM6A*, *KIT*, *KRAS*, *MLL*, *MPL*, *MYD88*, *NOTCH1*, *NPM1*, *NRAS*, *PDGFRA*, *PHF6*, *PTEN*, *PTPN11*, *RAD21*, *RUNX1*, *SETBP1*, *SF3B1*, *SMC1A*, *SMC3*, *SRSF2*, *STAG2*, *TET2*, *TP53*, *U2AF1*, *WT1*, and *ZRSR2*. The software used for analysis was Variant Interpreter (Illumina; Version 2.4.2.2846).

## Results

The demographic data of our population and full blood count results at study inclusion are summarised in ESM 2 and ESM 3. All patients, bar one, had a bone marrow examination as part of their investigations, and mutation analysis had consequently been carried out on bone marrow in 16 of the participants. Dysplasia in excess of 10% in one or more cell lines was reported on the bone marrow in 11 patients and there was no evidence of other bone marrow disease in any of the specimens. Four patients had a diagnosis of ICUS, while the remaining 12 patients had received a diagnosis of MDS. One patient had refused a bone marrow, so had mutation analysis on peripheral blood. This patient was an 81-year-old lady who was being monitored for persistent anaemia. She had an abnormal blood film with dysplastic features in the red cells and neutrophils, as well as notable platelet anisocytosis. None of the patients had been found to have a cytogenetic or a molecular abnormality when tested.

All participants completed the health questionnaire. Relevant data for each patient is displayed in ESM 4. Risk factors for copper deficiency were identified in ten patients, but none of these patients had copper levels below the reference range (ESM 5). One patient was found to have a low serum copper level, marginally below the reference range (11.3 μmol\L–reference range 12 μmol/L to 20 μmol/L). This was a 79-year-old Indian male who was being monitored for pancytopenia with dysplastic changes (reported to be in excess of 10%) in the erythroid and megakaryocytic lineages. He had been given a diagnosis of MDS. The patient did not report any risk factors for copper deficiency and there was no other comorbidity to suggest this diagnosis. The patient was offered copper replacement but failed to return to clinic for a prescription.

Interestingly, six patients (33%) had copper levels which were mildly above the reference range. Inflammation has been documented to produce a reactive rise in plasma copper level [17], and we therefore tested levels of C-reactive protein (CRP) to clarify the significance of these results. Two patients had elevated CRP levels at the time of testing (patient 14–CRP 32.5 mg/L; patient 15–CRP 58.8 mg/L; reference range 0–5 mg/L), so the elevated copper level in these patients is likely to be spurious. Four patients had CRP levels that were within the reference range (patients 12, 13, 16 and 17; CRP values 1.4, 0.3, 3.9 and 2.4 mg/L, respectively). The marginally elevated copper level in these patients remains unexplained.

## Discussion

Measuring copper levels has been reported as an important part of the workup when investigating patients with unexplained cytopenias [13]. The burden of misdiagnosing copper deficiency as MDS may be significant for both the patient and the healthcare provider. While studies in patients with a history of bariatric surgery and with neurological manifestations did include patients with cytopenia(s) [6, 14], our study is the first to evaluate the prevalence of copper deficiency in a population of patients presenting with predominantly haematological manifestations to a haematology clinic. The majority of our patients fulfilled WHO diagnostic criteria for MDS, and all lacked a genetic or molecular marker of clonality.

Myelodysplasia is an uncommon condition with only 1524 new cases reported nationwide in England in 2017 [18]. Our study has limitations, particularly its small size and single-centre recruitment design. In addition, we are unable to report on the response to copper replacement in our one patient who was found to have low levels because he failed to engage further with our services. On the other hand, our study has strength in its partially prospective design and the inclusion of a health questionnaire to screen for unanticipated risk factors.

Our results suggest that copper deficiency is uncommon in this population of patients, although one must be cautious in drawing conclusions due to the small size of our population. We were not able to demonstrate a relationship between the identification of risk factors for copper deficiency and copper levels, and this is almost certainly because of the low number of positive findings. It is not likely that the low copper level in one of our patients was contributing to his haematological features because his copper level was only a fraction outside the reference range, and he had a normal CRP (0.6 mg/L) at the time of testing. This suggests his copper level was not falsely raised to mask a more severe deficiency.

While copper deficiency should be considered in the differential diagnosis of cytopenias and dysplasia, our findings lead us to believe that indiscriminate testing of copper levels in patients with unexplained cytopenia may not be cost-effective. Based on data from larger studies, the identification of risk factors for the condition and signs of deficiency should improve the likelihood of a positive diagnosis [7, 9, 14]. We would recommend the consideration of copper deficiency in the differential of persistent cytopenias, and we suggest a risk-assessed approach to testing that seeks to identify patients who may be more likely to have this rare diagnosis.

## Electronic Supplementary Materials

ESM 1 (PDF 865 kb).

## Data Availability

All data generated or analysed during this study are included in this published article (and its supplementary information files).
