# Supplementary material for: Copper Levels in Patients with Unexplained Dysplastic Cytopenia
Source: Biol Trace Elem Res. 2020 Jun 2;199(3):825–8. doi: 10.1007/s12011-020-02203-x (PMC7813699; doi:10.1007/s12011-020-02203-x)
Supplement: Supplementary file 1 — (PDF 865 kb). [file 12011_2020_2203_MOESM1_ESM.pdf]

Biological Trace Element Research

Copper Levels in Patients with Unexplained Dysplastic Cytopenia

Dominic Butcher[1], Simona Deplano[2], Thomas Lofaro[3]

[1]Imperial College London, Department of Medicine, South Kensington, London, UK
[2]Imperial College NHS Healthcare Trust, Department of Haematology, Hammersmith Hospital, Du Cane Road, London, UK
[3]Imperial College NHS Healthcare Trust, Department of Haematology, Hammersmith Hospital, Du Cane Road, London, UK. ORCID ID: 0000-0003-1248-9192
Corresponding Author
Dominic Butcher
Email: dominic.butcher15@imperial.ac.uk
ORCID ID: 0000-0002-0812-5639

Supplementary Materials

ESM1 Inclusion and exclusion criteria

Inclusion criteria	***Exclusion criteria***
Patient had one or more persistent cytopenia(s) or macrocytosis	Bone marrow blasts found to be increased in excess of >5%
Patient had had a bone marrow examination performed to investigate persistent and unexplained cytopenia(s) or macrocytosis, and a diagnosis of ICUS, IDUS or MDS was made	Patient found to have a clonal cytogenetic or molecular abnormality (with the exception of deletion of chromosome Y)
Patient was over 18 years of age.	Patient had been started on treatment for MDS (excluding transfusion, erythropoietin and/or steroid therapy).
Patient had given consent to participate.	Patient unable or unwilling to give consent.
Patient was under the care of haematology at Hammersmith Hospital.	Patients who received copper replacement therapy in the past 12 months.

ESM2 Participant demographics

Demographic		***Frequency (%)***
Age		
	50 to 59	2 (12%)
	60 to 69	2 (12%)
	70 to 79	6 (35%)
	80 to 89	7 (41%)
Sex		
	Male	9 (53%)
	Female	8 (47%)
Ethnicity		
	Black Caribbean	4 (24%)
	Greek	1 (6%)
	Indian	1 (6%)
	Middle Eastern	2 (12%)
	Moroccan	1 (6%)
	Turkish	1 (6%)
	White British	5 (29%)
	White Irish	2 (12%)
BMI		
	<18.5	2 (12%)
	18.5 to 24.9	6 (35%)
	25.0 to 29.9	6 (35%)
	>30.0	3 (18%)

ESM3 Full blood count results at time of inclusion. All participants had at least one cytopenia M – males; F – females

Blood test	*Mean value (median)*	*Range*	*(Reference Range)*
Red cell count	*M:* 4.17 (3.95)<br>*F:* 3.38 (3.49)	*M:* 2.85 to 6.05<br>*F:* 2.32 to 4.61	(*M:* 4.7 to 6.1 $x10^{12}$/L)<br>(*F:* 4.2 to 5.4 $x10^{12}$/L)
Haemoglobin	*M:* 115 (118.0)<br>*F:* 104.3 (107.5)	*M:* 95 to 127<br>*F:* 74 to 135	(*M:* 130 to 180 g/L)<br>(*F:* 114-165 g/L)
Mean Cell Volume	92.6 (96.4)	58.9 to 109.9	(83.5 to 99.5 fL)
Platelets	127.2 (113.0)	34 to 387	(130 to 370 $x10^9$/L)
White Cell Count	4.6 (4.1)	2.2 to 8.1	(4.2 to 10.6 $x10^9$/L)
Neutrophils	2.2 (2.6)	0.6 to 6.0	(2.0 to 7.1 $x10^9$/L)

ESM4 Findings for each patient BMI – Body Mass Index, Cu – serum copper level, Hb – haemoglobin, RCC – red cell count, MCV – mean cell volume, WCC – white cell count, Neut – neutrophils, Plts – platelets, CRP – C-reactive protein, PPI – proton pump inhibitor

Patient	***Gender***	***Age***	***BMI***	***Cu***	***Hb***	***RCC***	***MCV***	***WCC***	***Neut***	***Plts***	***CRP***	***Dysplastic lineage(s)***	***Risk factors***
1	Male	79	21.30	11.3	113	6.05	58.9	2.9	1.5	36	0.6	Erythroid & Megakaryoblastic	
2	Male	78	28.10	13.3	119	5.74	62.4	3.6	1.4	133	1.0	Erythroid	
3	Male	81	31.60	14.7	127	4.47	82.7	4.1	0.7	116	No CRP	Trilineage	
4	Female	71	24.20	15.3	114	3.44	104.9	4.1	2.5	68	0.3	Erythroid & Megakaryoblastic	Dialysis, PPI
5	Male	82	32.50	16.8	124	4.15	101.0	4.2	2.8	126	2.5	Erythroid	Dialysis, PPI
6	Female	80	21.00	17.1	95	3.04	97.0	8.1	5.0	387	2.0	Declined marrow	
7	Male	67	27.40	18.9	104	3.45	92.0	4.9	2.2	194	No CRP	Trilineage	Low copper diet
8	Male	64	34.30	19	110	3.60	96.4	7.7	6.0	82	9.8	Erythroid	PPI
9	Female	84	17.90	19.5	111	3.65	101.4	2.3	1.6	87	1.1	Trilineage	PPI
10	Female	76	17.90	19.7	135	4.61	89.2	6.0	3.4	111	0.4	Megakaryoblastic	PPI, low copper diet
11	Male	83	21.10	19.7	118	3.25	107.7	3.2	2.2	151	2.9	Erythroid	
12	Female	55	27.18	20.6	81	2.32	109.9	4.0	2.0	34	1.4	Erythroid & Megakaryoblastic	
13	Male	83	27.40	20.9	125	3.95	95.9	5.7	2.8	113	0.3	Myeloid	PPI, multivitamins
14	Male	84	28.30	21.8	95	2.85	103.5	2.3	0.9	76	32.5	Myeloid & Megakaryoblastic	Multivitamin, neuropathy
15	Female	75	28.60	22.0	74	2.52	82.5	2.2	0.6	49	58.8	Trilineage	PPI
16	Female	59	20.10	22.8	104	3.88	83.0	8.0	4.6	200	3.9	Trilineage	
17	Female	72	20.80	23.6	121	3.54	105.4	5.3	3.7	199	2.4	Megakaryoblastic	

ESM5 Risk factors for copper deficiency in our population

Risk factor	***Frequency (%)***
Dialysis	2 (12%)
Proton pump inhibitor use	7 (41%)
Nutrition & Metabolic	
Low copper intake	2 (12%)
Alcohol excess	0 (0%)
Zinc-containing supplements	2 (12%)
Enteral nutrition, Total parenteral nutrition	0 (0%)
Copper metabolism defects e.g. Menkes, Wilson's	0 (0%)
Gastrointestinal	
Upper bowel resection, Bariatric surgery	0 (0%)
Short bowel syndrome	0 (0%)
Chronic diarrhoea	0 (0%)
Inflammatory bowel disease	0 (0%)
Neurology	
Optic neuropathy	1 (6%)
Peripheral neuropathy	1 (6%)

ESM6 Health Questionnaire used to gather patient information

Imperial College Healthcare NHS
NHS Trust

Imperial College London

Health Questionnaire

For the participants of the study: Reviewing the prevalence of copper deficiency in patients with suspect myelodysplastic syndromes

Please answer as many questions as you wish. The information you provide will only be available to researchers in this study, who will anonymise this information when it is entered into a password-protected document on an Imperial College Healthcare NHS Trust computer. This form will be disposed of in a confidential waste bin.

Full name: ____

Date of birth: ____

Gender: ____

Occupation: ____

Are you exposed to any hazardous chemicals at work? Yes/No. If yes, please specify:

____

Ethnic Origin:

This is not about nationality, place of birth or citizenship. It is about the group to which you perceive you belong. Please circle the appropriate answer.

Prefer not to say

White: English/Welsh/Scottish/Northern Irish/Irish/British/Other, please specify: ____

Mixed/multiple ethnic groups: White and Black Caribbean/White and Black African/White and Asian/Other mixed background, please specify:

____

Asian: Indian/Pakistani/Bangladeshi/Chinese/Asian British/Other, please specify: ____

Black: Black/African/Caribbean/Black British/Other, please specify:

____

Any other ethnic group, please specify:

____

Height: ______________________________ **Weight:** _________________________

Current medications:

____

____

____

____

Are you currently undergoing any form of dialysis? Yes/No. If yes, please give details and the length of time you have been on dialysis:

____

____

Are you currently involved in any other research studies? Yes/No. If yes, please give details of the other study/studies:

____

____

Diet:

Are you vegetarian/vegan (please delete as appropriate)? Yes/No.

Do you follow a specific diet plan? Yes/No. If yes, please give details of your diet:

____

____

____

____

Do you take any vitamins or supplements e.g. zinc supplements? Yes/No. If yes, please specify:

____

____

____

Have you received enteral nutrition (i.e. received food through a tube that is attached directly to your intestinal tract) or intravenous nutrition in the past 3 months? Yes/No. Please specify why you had this type of nutrition and for how long:

____

____

____

How much alcohol do you drink in a typical week?

None (please circle if appropriate)

Beer _____ pints

Cider _____ pints

Wine _____ bottles

Spirits _____ bottles

For how many years has this level of drinking been typical?

____

Medical History:

Have you ever had gastrointestinal surgery e.g. bowel resection, bariatric surgery? Yes/No. If yes, please specify:

____

____

Do you suffer from any of the following?

Coeliac Disease. Yes/No.

Cystic Fibrosis. Yes/No.

Short Bowel Syndrome. Yes/No.

Chronic Diarrhoea. Yes/No

Any other digestive or malabsorption condition (please specify):

____

____

Peripheral neuropathy. Yes/No.

Myeloneuropathy. Yes/No.

Spasticity. Yes/No.

Any other neurological condition (please specify):

____

____

Any visual impairment (please specify):

____

____

Wilson's Disease. Yes/No.

Menkes Disease. Yes/No.

Have you ever been diagnosed with any other conditions of copper metabolism? Yes/No. If yes, please specify:

____

____

Many thanks for any information you have provided and for your participation in our study.
